# Supplementary material for: From sequence to enzyme mechanism using multi-label machine learning
Source: BMC Bioinformatics. 2014 May 19;15:150. doi: 10.1186/1471-2105-15-150 (PMC4229970; doi:10.1186/1471-2105-15-150)
Supplement: Additional file 2 — Java code of ml2db. Additional file ml2db_code.tar.gz contains the Java source code to run the multi-label machine learning experiments and save the results to database. The code’s Javadoc is included. [file 1471-2105-15-150-S2.zip › additional file 2/ml2db/ml2db/doc/index-files/index-6.html]

F-Index


JavaScript is disabled on your browser.


- Overview
- Package
- Class
- Use
- Tree
- Deprecated
- Index
- Help

- Prev Letter
- Next Letter

- Frames
- No Frames

- All Classes

A B C D E F G H I J K L M N O P Q R S T U V W X 


## F

FA\_ATT1 - Static variable in class test.ml2db.dataharness.DataOne


FA\_ATT2 - Static variable in class test.ml2db.dataharness.DataOne


FA\_ATT3 - Static variable in class test.ml2db.dataharness.DataOne


FA\_ATT4 - Static variable in class test.ml2db.dataharness.DataOne


FA\_ATT5 - Static variable in class test.ml2db.dataharness.DataOne


FALSE\_NEGATIVES - Static variable in class main.ml2db.mulan.predict.PredictionsSaver


FALSE\_POSITIVES - Static variable in class main.ml2db.mulan.predict.PredictionsSaver


FalseNegatives - Class in main.ml2db.mulan.learn.evaluation
:   Class

FalseNegatives(int) - Constructor for class main.ml2db.mulan.learn.evaluation.FalseNegatives


FalsePositives - Class in main.ml2db.mulan.learn.evaluation
:   Class

FalsePositives(int) - Constructor for class main.ml2db.mulan.learn.evaluation.FalsePositives


FEATURE\_ATTRIBUTES\_TAG - Static variable in class main.ml2db.weka.InstAttValueMap


FIG - Static variable in class main.ml2db.graphviz.GraphViz


FIGURES\_PATH - Static variable in class main.ml2db.ProjectParameters


fillArff() - Method in class main.ml2db.weka.Arff
:   Loads instances, but does not write the arff file or arff database
    record.

fillArff() - Method in class main.ml2db.weka.DataSetManager


fillDot(IdMultiLabelInstances) - Method in class main.ml2db.graphviz.GraphVizArff


fillInstance(String, SparseInstance) - Method in class main.ml2db.weka.InstancesFiller
:   Returns an instance filled with all its attribute values.

fillInstances() - Method in class main.ml2db.weka.DataSetGenerator
:   If size is judged massive for the current hardware, while filling, write
    instances one by one to temp file.

fillMetrics(List<Measure>) - Method in class main.ml2db.mulan.measuremetrics.PredictionsPerformanceMeasurer


FILTER\_OLD\_TEST\_SET - Static variable in class test.ml2db.dataharness.TestProjectParameters


FILTER\_TRAIN\_SET - Static variable in class test.ml2db.dataharness.TestProjectParameters


find(double[]) - Method in class main.ml2db.al.utility.confidence.ConfidencesStorage


findADistance(String, String) - Method in class main.ml2db.al.utility.globaldistance.DistancesStorage


findBySparseAttribString(String) - Method in class main.ml2db.al.utility.neighbours.NeighbourhoodNetwork
:   public static void main(String[] args) { int arffId = 13; String filePath
    = ProjectParameters.PREDICTIONS\_PATH + arffId + "\_plants" +
    TimeUtils.getTimeStampForFileName() + ".dot";
    try { IdMultiLabelInstances plants = new IdMultiLabelInstances(arffId,
    ProjectParameters.getRemoteDbManagers()); NeighbourhoodNetwork network =
    new NeighbourhoodNetwork(plants); GraphVizPrinter
    .printNeighboursForGraphviz(filePath, network, false);
    } catch (InvalidDataFormatException e) { // TODO Auto-generated catch
    block e.printStackTrace(); }
    }

findByWekaHashCode(int) - Method in class main.ml2db.al.utility.neighbours.NeighbourhoodNetwork


Folds - Class in main.ml2db.mulan.learn.crosseval
:   Stores and delivers folds for cross validation

Folds(IdMultiLabelInstances, int) - Constructor for class main.ml2db.mulan.learn.crosseval.Folds


fromEnumerationToArrayList(Enumeration<Attribute>) - Static method in class main.ml2db.mulan.mulanarff.AttributeUtils


fromEnumerationToVector(Enumeration<Attribute>) - Static method in class main.ml2db.mulan.mulanarff.AttributeUtils


fromEnumerationToVector(Enumeration<Instance>) - Static method in class main.ml2db.mulan.mulanarff.InstanceUtils


fromWekaToIdInstance(Instance) - Method in class main.ml2db.mulan.predict.PredictionsSaver
:   Get an identified instance corresponding to this weka instance (same
    attributes)

fullRun(DbManagers, boolean, boolean, boolean, boolean, int, int, MulanLearner) - Static method in class main.ml2db.mulan.learn.traintest.TrainTestFullRun
:   Executes: 1.

A B C D E F G H I J K L M N O P Q R S T U V W X

- Overview
- Package
- Class
- Use
- Tree
- Deprecated
- Index
- Help

- Prev Letter
- Next Letter

- Frames
- No Frames

- All Classes
